# Pre-clinical evaluation of soybean-based wound dressings and dermal substitute formulations in pig healing and non-healing *in vivo* models

**DOI:** 10.4103/2321-3868.143624

**Published:** 2014-10-25

**Authors:** Rostislav V. Shevchenko, Matteo Santin

**Affiliations:** 1 Pharmidex Pharmaceutical Services Ltd, London, UK; 2 Brighton Centre for Regenerative Medicine, University of Brighton, Huxely Building Lewes Road, Brighton, BN2 4GJ UK

**Keywords:** Dermal substitutes, *in vivo* models, wound dressings, soyabean-based biomaterials

## Abstract

In the last decade, a new class of natural biomaterials derived from de-fatted soybean flour processed by either thermoset or extraction procedures has been developed. These biomaterials uniquely combine adaptability to various clinical applications to proven tissue regeneration properties. In the present work, the biomaterials were formulated either as hydrogel or as paste formulation and their potential as wound dressing material or as dermal substitute was assessed by two *in vivo* models in pig skin: The healing full-thickness punch biopsy model and the non-healing full-thickness polytetrafluoroethylene (PTFE) chamber model. The results clearly show that collagen deposition is induced by the presence of these biomaterials. A unique pattern of early inflammatory response, eliciting neutrophils and controlling macrophage infiltration, is followed by tissue cell colonization of the wound bed with a significant deposition of collagen fibers. The study also highlighted the importance in the use of optimal formulations and appropriate handling upon implantation. In large size, non-healing wounds, wound dermis was best obtained with the paste formulation as hydrogels appeared to be too loose to ensure lasting scaffolding properties. On the contrary, packing of the granules during the application of paste reduced biomaterial degradation rate and prevent the penetration of newly vascularized tissue, thus impeding grafting of split-thickness autologous skin grafts on the dermal substitute base.

## Introduction

Bioengineered products able to support the healing of chronic ulcers, deep wounds and burns are in increased demand to improve patients’ quality of life and survival rate. Many different biomaterials have been investigated for the role of dermal substitutes[1,2] and no material has yet been described that fulfills all the ideal features.

**Table 1:** 

Access this article online	
**Quick Response Code**:	**Website**: www.burnstrauma.com
	**DOI**: 10.4103/2321-3868.143624

The clinical performance of wound dressings and dermal substitutes based on synthetic biomaterials is limited to the ability of these polymers to keep the wound environment moist, to protect the wound site from infections and in some cases to encourage cell colonization.[3–6] Natural polymers of human and animal origin bear biochemical features that can favor the healing processes. Indeed, biomaterials based on protein or polysaccharide composition such as collagen, glycosaminoglycans, agarose, alginate, chitosan and fibrin glue have been shown to be able to promote interactions with biochemical and cellular components leading, for example, to hemostatic functions (*e.g.*, alginate and fibrin) and to bioligand-driven cell activity control (*e.g.*, collagen and hyaluronan). However, the use of biopolymers in regenerative medicine is limited by their antigenic potential, by the risks of transmittable diseases, by batch-to-batch variability and, in some cases, by their relatively high extraction costs.

For example, collagen-based materials have been investigated more extensively as scientists tried to produce dermal substitutes mimicking normal skin where dermal collagen plays very important roles in structure and function. Initially, raw human or animal skin, either fresh or frozen, was used to cover wounds but immunological reactions were soon observed leading to poor experimental and clinical performances.[7] These immunological reactions were attributed to the presence of cellular component and led to the use of decellularized dermal sheets. These showed no rejection upon implantation while providing solid dermal wound bed for the later application of split-thickness skin grafts.[7–9] Although this approach has been clinically successful, it still brings drawbacks that need to be resolved. First, there is a problem of transmissible diseases. Although donors are screened extensively and manufacturing processes minimize such a possibility, risks still exist. Ethical issues have also to be considered as important for some religious groups.[10] Furthermore, the logistics, suitable donor selection, coordination of skin banks networks and the storage of human-derived products are all major issues associated with this approach and limiting its use. Dermal substitutes of non-human origin like Integra®, Matriderm® and Terudermis®, although still expensive to produce and store, are less subject to the ethical and cross-contamination issues since animals have an interspecies resistance to some viral disease.

In the last decade, a new class of natural biomaterials derived from de-fatted soybean flour processed by thermoset or extraction procedures has been developed.[11] These biomaterials uniquely combine adaptability to various clinical applications to clear tissue regeneration properties. Formulations can be easily prepared that span from films and granules to hydrogels and pastes or bioglues. *In vitro* studies on fibroblasts, osteoblasts and macrophages as well as *in vivo* studies of bone regeneration have clearly demonstrated the healing properties of these biomaterials.[12–16]

These biomaterials need to be distinguished from those based on soy protein only.[17,18] Soy protein scaffolds, for example, have been fabricated using freeze-drying and 3D printing and tested for their biocompatibility *in vivo* by a subcutaneous implant model in mice.[19] The study compared the soy protein with bovine collagen in terms of acute and humoral immune response showing that soy scaffolds fully degraded after 14 days with no sign of fibrosis as opposed to collagen scaffolds that remained intact for 56 days. Immunological analysis showed no soy-specific IgE indicating the absence of allergic response to the soy implants. Likewise, a rat wound-dressing model of partial-thickness skin wounds was used to study newly developed chitosan/soy-based membranes as wound dressing materials showing high healing properties of these materials when compared to a commercially available dressing such as Epigard®.[20]

This paper shows for the first time the healing properties of soybean-based hydrogels and pastes in 2 pre-clinical *in vivo* models based on healing (full-thickness punch biopsy model) and non-healing (full-thickness chamber model) wounds in pigs.

Porcine animal models have been widely utilized for wound healing and skin regeneration studies as the most similar to the relative clinical applications.[21] The use of punch biopsies to create experimental wounds has been described for several animal models, including guinea pig for skin studies.[22] Punch biopsy models in pigs were also used for skin wound healing studies.[23–25] A percutaneous polytetrafluoroethylene (PTFE) full-thickness wound chamber pig model has also been previously used in many studies such as those assessing biointegration of different types of biomaterials and skin cells and has shown excellent results.[26]

## Materials and methods

### Material preparation

Soybean-based biomaterials were prepared from de-fatted soybean flour as previously described.[12] Briefly, soybean-based hydrogels were obtained by extraction of soybean protein, carbohydrate and isoflavone fraction through a 80/20% ethanol/water extraction process. The extracts were filtered, freeze-dried, reconstituted in a minimum volume of 0.1M CaCl_2_ solution to obtain a hydrogel. Granules of soybean-based biomaterials were obtained by thermosetting of soybean curd at 60°C for 20 h. The thermoset material was then grinded in a blender and granule size selected by filtering through stainless steel filters to achieve a controlled granule diameter within the 215–300 µm range. Two paste formulations were obtained mixing granules with hydrogels without or with genipin cross-linking as it follows:

1. Soybean-based granules (40%) and soybean-based hydrogel (60%).
2. Soybean-based paste cross-linked with 1% (w/v) genipin.

**Figure 1:**
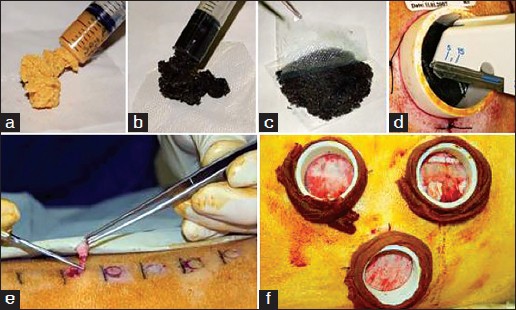
Soybean-based dressing formulations. (a) Non-cross-linked paste (60% by weight soybean thermoset granules and 40% by weight hydrogel), (b) genipin cross-linked paste, (c–d) typical application procedure on pig model, (e) punch biopsy model, (f) PTFE chamber model.

Figures 1a–d show typical soybean paste non-cross-linked [Figure 1a] and cross-linked paste [Figure 1b] formulations and a typical application to PTFE chamber model [Figures 1c and d].

### *In vivo* Experiments

Two types of wound models were adopted [Figures 1d and e]. All experiments were approved by a local ethical review committee and carried out according to the “Animals (Scientific Procedures) Act 1986” under Home Office project license PPL 80/1735 and personal license PIL 80/8828 granted on 1/10/2004.

### Full-thickness biopsy model

Three pigs were used for this study, and the total number of experimental wounds was 72, with 24 per animal. The study was carried out in two experimental stages. As a first stage, biointegration of biomaterials was assessed in one animal. The second stage included the assessment of the wound healing properties of soybean-based biomaterials in two animals. The healing process starts immediately after wounding but the most active proliferative phase occurs between 5 and 21 days with maximum effect between the 7^th^ and 14^th^ day. As a consequence, punch biopsies were taken at two time points: 8 and 15 days post-implantation. Sample evaluation of integrated materials at day 27 was performed to assess the relatively long-term performance of the applied materials. A 10 mm punch was used to harvest biopsies. Upon removal, each biopsy was bisected vertically, one-half was processed to paraffin wax embedding by routine automated procedures and the other half was frozen for cryo-sectioning. Paraffin sections (5 µm) were stained with hematoxylin and eosin (HE) to reveal basic structure of the tissue, and with picrosirius red (PSR) for collagen deposition visualization. Birefringence images were also taken to study collagen orientation.

### Full-thickness chamber model

Seven large white/landrace crossbred pigs obtained from a single registered supplier were used for this study. A total of 42 experimental wounds were created with 6 per animal. Wounds were treated with soybean-based biomaterials in addition to a number of alternative biomaterials (data published elsewhere).

Two circular skin grafts 5 cm in diameter and 500 µm thick were harvested and reapplied to the excised wound serving as a control treatment group. The epithelial layer was trimmed with a Zimmer air dermatome (setting 3, 6/1000th of an inch) before dermal harvest was performed. Fat was trimmed off the wound bed with a scalpel and dermis was reapplied to the wound and sutured in place.

Negative control wounds did not receive any specific treatment and were left to form a layer of granulation tissue.

In a preliminary study, two pre-measured 1.3 ml volumes of soybean-based hydrogels were spread evenly by spatula on top of the wound to form a layer approximately 1 mm thick. The lack of consistency and long-term stability of the hydrogel prompted the development of two new soybean-based paste formulations that were tested in combination to split-thickness autologous skin grafts.

Paste (1.3 ml) was extruded from a syringe, and a circular layer of approximately 5 cm in diameter and 1 mm thick was then formed with a spatula.

Split-thickness autologous skin graft approximately 5 cm × 5 cm × 0.015 cm was harvested with an air-driven Zimmer air dermatome. The graft was trimmed to a circular shape and was applied to granulating wounds at day 0 or 6 (positive control) or applied over dermal substitute biomaterials.

Following biomaterial implantation and skin graft fixing by Appose® and Autosuture® skin staples, each chamber was dressed with a double layer of Telfa Clear® fenestrated non-adherent silicone dressing.

To use the chamber model more effectively and to reduce number of animals, serial intrachamber punch biopsies were taken with a final whole wound excision biopsy. Taking such biopsies at different time points allowed assessment of the time of biointegration and the most appropriate time for split-thickness skin grafting for each biomaterial based on progression of the neovasculature network formation.

Wound dressings were changed every 3 days with simultaneous clinical wound assessment. Full-thickness wound excision biopsies were done at day 3, 6 and 14. Three 5.0 mm punch biopsy samples (top, middle and bottom of the wound) within each chamber were taken to assess material integration. The same procedure was repeated on day 4 and 7. On day 14, wounds were harvested by excision and 10 mm biopsies were taken within harvested tissue samples. Upon removal, each biopsy was bisected vertically, one-half was processed to paraffin wax embedding by routine automated procedures and the other half was frozen for cryo-sectioning. Paraffin sections (5 mm) were stained with HE to reveal basic structure of the tissue and with PSR for collagen deposition visualization. Cryo-sections were processed for immunohistochemical staining by incubation in a humidified chamber for 20 min with normal goat serum (Sigma-Aldrich Company Ltd., UK) and a further hour with the primary rabbit anti-human von Willebrand factor polyclonal antibody (anti-vWF, DakoCytomation, UK) used at a 1:40,000 final dilution to identify blood vessel formation. To study the epithelial cover, cryo-sections were also processed for immunofluorescent staining with mouse monoclonal anti-human cytokeratin 14 (clone LL002) antibody at a 1:50 dilution. Rabbit anti-human von Willebrand factor polyclonal antibody (1:200 concentrate) was used at 1:200 dilution to identify blood vessel formation. Macrophage/L1 Protein/calprotectin Ab-1 (clone MAC 387) antibody was used at 1:40 dilution for tissue macrophage visualization. Tissue sections were washed with PBS and incubated for one further hour with secondary antibody fluorescein-isothiocyanate (FITC)-labeled goat anti-rabbit IgG (MP Biomedicals, UK) at 1:100 dilution as recommended by the supplier.

The stained tissue sections were viewed and analyzed through a fluorescent microscope, Olympus BH2, and photographed within 24 h of staining using a Spot 1.1.0 digital camera (Diagnostic Instruments, Inc., USA) and Image-Pro Plus software (Media Cybernetics, Inc., USA). Digital photographs of histological tissue sections were loaded into Image-Pro Plus 4.0 image analysis software package (Media Cybernetics, Inc., USA) and a grid mask was superimposed over the image. Area, depths and host cellular migration and neovascularization of the implanted biomaterials were evaluated by counting elements of interest, either manually or automatically in 5 fields of view of each tissue section at magnification ×100 (cellular migration), ×40 (neovasculature in-growths) or ×20 (granulation tissue formation). Statistical analysis was performed in SigmaStat 3.5 (Systat Software, Inc., USA) using either t-test Student’s or one-way analysis of variance (ANOVA) with the further post-hoc analysis between groups (Tukey test). *P*-values with less than 0.05 between tested groups were considered to be statistically significant.

Clinical digital photographs of all experimental wounds where epithelial treatment was applied (*n* = 27) were loaded into Image-Pro Plus 4.0 image analysis software package (Media Cybernetics, Inc., USA), and the total area of the wound and the area of epithelialization were digitally traced and quantified. Epithelialization was expressed as a percentage ratio of the epithelialized area to the total wound area.

Tissue microphotographs were arranged into montage panels (each consisting of 15–50 individual images) to reveal the entire cross-section of the excised experimental wound. Levels of epithelialization, inflammation, neovascularization and granulation tissue formation were assessed at magnifications ×20, ×40, ×100 and ×200 either qualitatively or quantitatively with the help of an image analysis software. Obtained data were analyzed statistically using ANOVA with pair-wise multiple comparison of experimental groups (Tukey Test) and differences between groups were considered to be statistically significant at *P* < 0.05.

## Results

### Full-thickness biopsy model

**Figure 2:**
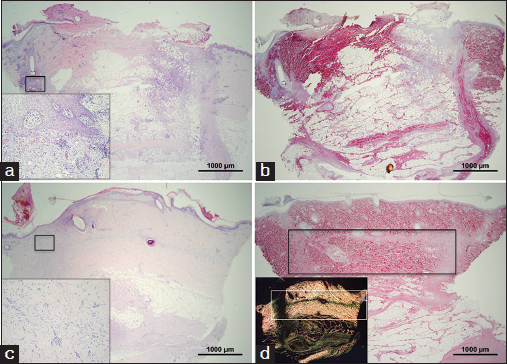
Histology of control wounds in the punch biopsy model (×20). (Scale bar=1000 µm) (a and b) Day 8 of healing (c and d) and day 15 of healing. Hematoxylin and eosin staining (a and c) and Picrosirius staining (b and d). (a and c) Inserts in (a) and (c) highlight high magnification (×200) images of area of inflammatory cell infiltration. (d) Insert highlights birefringence image showing newly deposited collagen fluorescing green at the interface with the graft.

The full-thickness biopsy model showed that control wounds rapidly healed with epithelialization as expected in this animal model. A reduction in acute inflammatory response was observed with time in all wounds [Figure 2a]. An inflammatory infiltrate was visible on day 8, but it was greatly reduced by day 15 [Figures 2a and b, inserts]. PSR staining also showed clear deposition of new collagen visible as small fibers fluorescing green by birefringence microscopy. This was shown by either a thin boundary of collagen birefringence [Figure 2c, rectangle] or clearly observed as a distinct area [Figure 2d, rectangle].

**Figure 3:**
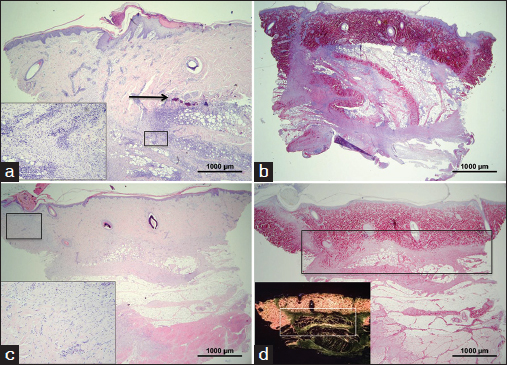
Histology of soybean-based hydrogels in the punch biopsy model (×20). (Scale bar=1000 µm) (a and b) Day 8 of healing (c and d) day 15 of healing. Hematoxylin and eosin staining (a and c) and Picrosirius staining (b and d). (a and c) Inserts in (a) and (c) highlight high magnification (×200) images of area of inflammatory cell infiltration. (d) Insert highlights birefringence image showing newly deposited collagen fluorescing green at the interface between the graft and the area of hydrogel implantation.

All the wounds treated with soy-containing biomaterials showed signs of inflammation after 8 days of implantation [Figure 3a] with the presence of the implanted hydrogel still visible within the wound site [Figure 3a, arrow]. This acute inflammatory reaction toward the soybean-based hydrogel was resolved by day 15 and the material resulted completely re-absorbed and tissue cells have populated the wound site depositing new extracellular matrix [Figure 3b]. The deposition of the new collagen was clearly showed by birefringence microscopy [Figure 3c] at a level comparable to that of the control [Figure 2d]. Experimental wounds treated with soybean-based hydrogel did not seem to resist contraction due to the lack of mechanical stiffness of the implanted hydrogel material.

### Full-thickness chamber model

No bacterial wound contamination or chamber extrusion was observed and therefore all wounds were made available for analysis.

Control wounds showed no epithelialization, neither by clinical visual inspection nor by histological analysis (data not shown).

**Figure 4:**
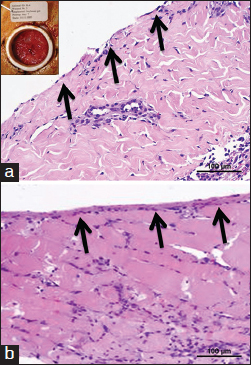
Soybean-based hydrogels in the PTFE chamber wound model after 2 days of implantation. (Scale bar=100 µm) (a and b) Histological analysis by hematoxylin and eosin staining of non-treated wound (a) and soybean hydrogel (b). Insert in (a) shows visual inspection of the wound. Arrows indicate a newly formed tissue layer deposited on the muscle layers.

In the preliminary study, soybean-based hydrogel tended to run slowly down the vertical surface of the wound although it remained in place when silicone dressing was applied (data not shown). The first dressing change procedure performed on day 2 revealed macroscopic absence of the applied soybean-based hydrogel material in the wounds. No soybean-based hydrogel was seen in histological sections from day 2 and thereafter and no direct biomaterial visualization was possible to quantify host cell penetration or scaffold neovascularization. Other detectable effects of soybean-based hydrogel application, like collagen deposition, were marginally increased when compared with control wounds [Figures 4a and b, arrows]. This relatively thin tissue layer was directly apposed on the muscle layer. Visual inspection of the wounds treated with soybean-based hydrogel showed granulation tissue formation at the sites of biomaterial implantation. The thickness of the granulation tissue layer proceeded from 0.26 mm (SD = 0.19) on day 4 to 1.19 mm (SD = 0.71) on day 7 [Figure 4b], and to 4.8 mm (SD = 3.24) on day 14 (data not shown).

**Figure 5:**
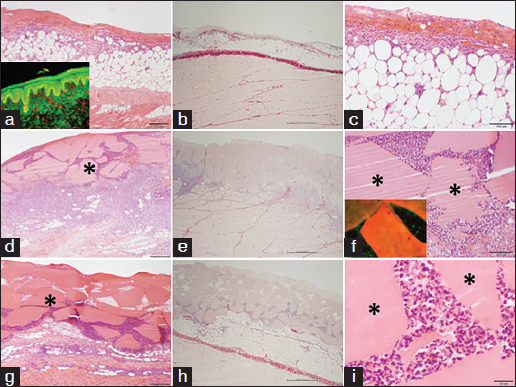
Soybean-based pastes in the PTFE chamber wound model after 3 days of implantation. (a–c) Control wound with applied split-thickness autologous graft, (d–f) non-cross-linked paste of Figure 1a, (g–i) cross-linked paste of Figure 1b. (b, e, h) Picrosirius staining, while all remaining images show hematoxylin and eosin staining at different magnifications. Insert in (a) shows keratinocyte immunostaining, insert in (f) shows immunostaining for macrophages (MAC387 antibody). Asterisks indicate soybean-based paste granules. Scale bar: a, d and g, 200 µm; b, e and h, 1000 µm; c and f, 100 µm;; i, 20 µm;.

For these reasons, further experiments were performed with the soybean-based paste that provided information for optimizing the handling properties of the material and its stability in the wound thus also giving the opportunity of evaluating its skin grafting substrate properties. Unlike the previously assessed soybean-based hydrogel formulation, the soybean-based paste biomaterial proved to persist in the wound during the course of the experiment [Figures 5d–i].

Control split-thickness skin grafts applied directly to the wound bed survived and integrated into the wound when applied as early as day 0. Histological assessment revealed well-structured epithelium with an undulating basal layer and multiple deep rete ridges [Figure 5a]. The epithelial nature of cells was confirmed by positive immunostaining for cytokeratin-14 [Figure 5a, insert]. Granulation tissue formation was minimal in these control wounds. The epithelialized area was 71.28%, SD = 17.19 on day 14. The influx of phagocytic leucocytes was relatively limited.

**Figure 6:**
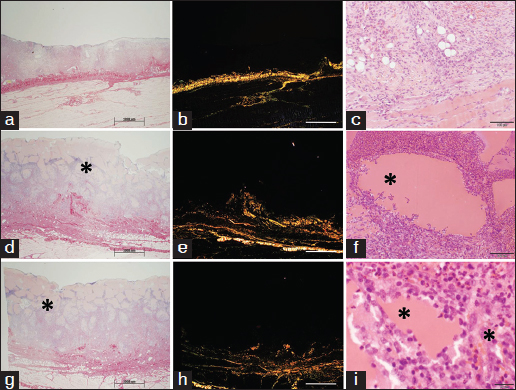
Soybean-based pastes in the PTFE chamber wound model after 6 days of implantation. (a–c) Control wound with applied split-thickness autologous graft, (d–f) non-cross-linked paste of Figure 1a, (g–i) cross-linked paste of Figure 1b. (a, d, g) Picrosirius staining, (b, e, h) Birefringence microscopy, (c, f, i) hematoxylin and eosin staining at high magnification showing polymorphonucleate infiltrates degrading paste granules. Asterisks indicate soybean-based paste granules. Scale bar: a, d and g, 200 µm;m; b, e and h, 1000 µm;m; c and f, 100 µm;m; i, 20 µm;m.

**Figure 7:**
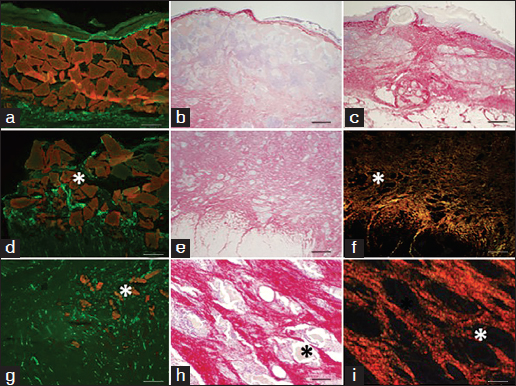
Soybean-based pastes in the PTFE chamber wound model after 14 days of implantation. (a–c) Typical soybean-based paste tissue regeneration profile throughout wound depth. (d–f) Typical soybean-based paste tissue regeneration profile close to the wound bed. (g–i) Typical soybean-based paste tissue regeneration profile showing details of new extracellular matrix deposition among degrading granules. (a, d, g) Von Willebrand immunostaining showing degree of neovascularization in paste with compacted granules (a) and dispersed granules (d, g). (b, c, e, h) Picrosirius staining, (f, i) birefringence microscopy. Asterisks indicate soybean-based paste granules. Scale bar: a, d and g, 500 µm;m; b and e, 1000 µm;m; c and f, 1000 µm;m; h and i, 500 µm;m.

The soybean-based paste was clearly visible in the wound clinically (data not shown) as well as histologically from day 2 to 14 [Figures 5–7]. On day 2, HE staining clearly showed the prevalence of polymorphonucleate cells gradually infiltrating the material granules from the bottom of the wound upward and degrading them both in non-cross-linked and cross-linked pastes [Figures 5d, f, g, i, asterisks]. MAC387 staining clearly show that the presence of macrophages was very limited [Figure 5f, insert]. The deposition of new tissue observed on day 2 was negligible [Figure 5e and h] and the split skin graft did not graft onto the biomaterial bed in most of the cases [Figures 5d and g].

This pattern gradually evolved on day 6 when control split graft showed no significant improvement of integration [Figures 6a–c], while polymorphonucleates progressed toward the top of the wounds treated with both non-cross-linked and cross-linked soybean-based pastes [Figures 6d and g]. However, deposition of new collagen gradually infiltrating the granules from the bottom of the wound upward was clearly observed by both PSR staining and birefringence microscopy [Figures 6d, e, g, h]. The latter highlighted relatively thick fibers (orange birefringence), which is typical of more mature collagen. This newly formed extracellular matrix appeared to replace the granulocyte-rich infiltrates. Where still present, these cells lead to a more pronounced degradation of the biomaterial granules [Figures 6f and i].

This pattern of healing was even more clearly observed on day 14 [Figures 7a–i]. At this point, experimental wounds with applied soybean-based paste revealed two distinct patterns of vascularization; soybean-based paste showing relatively compacting of granules did not show vascular in-growth deeper than 200–300 µm; [Figure 7a], whereas wounds with dispersed soybean-based particles were infiltrated by new microvasculature throughout their thickness [Figures 7d and g]. The degree of collagen deposition followed the same pattern with the less compacted granules allowing a full penetration of new extracellular matrix [Figures 7b and c]. High magnification histology clearly showed the infiltration of collagen fibrils throughout the granules and within their degrading structure [Figure 7h], while birefringence clearly showed their ordered structure [Figure 7i].

## Discussion

Skin damages generated by pathologies or trauma require the support of wound dressing and dermal substitutes capable of encouraging tissue regeneration while protecting the wound bed from infections and dehydration. Unless expensive and potentially carcinogenic growth factors are uploaded in the hydrogel mesh,[27] wound dressings are of relatively simple composition and design as they are primarily engineered to keep the wound moist, oxygenated and protected by external microbial insults. For these reasons, wound dressings are mainly used in the treatment of chronic foot and leg ulcers as well as in the case of burns of limited area surface. Their ability to promote healing is very limited in the case of chronic ulcers as those occurring in diabetic patients or in the case of pressure ulcers as those caused by long-term dwelling in static positions.

Dermal substitutes (acellular and cellularized) have been made available to clinicians to intervene on large-area wounds and burns.[28]

Recent investigations about the use of soybean as a potential source for biomaterials have highlighted that these biomaterials in the form of films, granules, hydrogel or paste exert several specific bioactivities on various types of tissue cells and promote bone regeneration *in vivo*.[14,15] Previous studies have also shown that these tissue regeneration properties can be ascribed to the unique combination of the scaffolding properties of these biomaterial and presence of isoflavones, plant estrogens capable of selectively interacting with the estrogen receptor (ER) β of the nuclear membrane rather than the ERa, thus activating cell differentiation rather than proliferation with a consequent induction of the production of extracellular matrix components, that is, collagen.[12,16] Interestingly, it is known that skin is the largest non-reproductive system target for estrogens and their effect is executed through nuclear estrogen receptors ERa and ERβ.[29] ERβ is particularly widespread in skin and expressed in basal keratinocytes, bulge region of hair follicles and papillary dermis.[30,31] The lack of estrogens results in skin thickness reduction, skin dryness, loss of elasticity and poor wound healing.[29,32,33] Activation of ERa and ERβ keratinocyte receptors as well as reported transactivation of epidermal growth factor (EGF) receptor by estrogens[34] may explain estrogen-induced keratinocyte proliferation.[35,36] Estrogens were found to reduce apoptosis, induced by the oxidative stress in human keratinocytes.[37] Estrogens also exhibit their effect on dermal component of the skin by increasing fibroblasts proliferation. These observations prompted the pre-clinical validation of the soybean-based biomaterials when formulated as hydrogel or as paste (mix of thermoset granules and hydrogel) and to the purpose of using them either as wound dressing material or as dermal substitute to support split-thickness skin grafts in burns or other types of extended skin loss.

The use of two *in vivo* models of skin regeneration in pigs clearly highlighted the ability of these natural biomaterials to reduce a chronic inflammatory response driven by macrophages common to many other biomaterials and to stimulate the deposition of collagen during the healing process. These findings confirmed previous *in vitro* studies showing the inhibition of macrophages and the stimulation of fibroblast collagen synthesis by these biomaterials.[12,16] In particular, a healing model such as the full-thickness punch biopsy showed that soybean-based hydrogels of a relatively loose consistency are suitable for the treatments of relatively small and deep defects such as those of chronic foot ulcers. The fluidity of the biomaterial allow its in-depth penetration into the wound bed where it stimulates the acute inflammation in the early phase and stimulate a gradual deposition of new extracellular matrix where collagen fibers acquire an orientation similar to that of the healthy dermis. The fluidity of the hydrogel also support wound contraction, a process that is fundamental to wound closure.

However, the same hydrogel formulation was shown to be not suitable for the treatment of large defects as it was not able to offer sufficient and stable scaffolding role for the regenerating tissue. Toward this end, a soybean paste consisting of a mix of granules derived from the thermosetting of soybean curd and of the same hydrogel was used as such or further reinforced by cross-linking with a natural dialdehyde, the genipin. These new paste formulations were used in the non-healing full thickness chamber model where a PTFE ring prevents tissue in-growth from the periphery of the wound bed and allows regeneration only from its bottom. The experiments clearly showed that the two types of pastes could ensure stability and tissue scaffolding properties over a longer period of time as they were still present after 25 days of implantation. Uniquely, the fast degradation of the hydrogels was creating space among the more stable granules that gradually encouraged tissue infiltration, first in form of acute inflammatory response exhibiting a prevalence of neutrophils to that of macrophages, later inducing the thorough invasion of new dermis where collagen and vessels gradually progressed from the bottom of the wound toward its surface. However, the data also showed that the optimal formulation of the paste is as important as its biocompatibility and tissue regeneration properties. In fact, when granules were excessively packed in the wound bed during the surgical procedure, tissue in-growth was delayed or even substantially inhibited. This was detrimental not only for the process of infiltration of newly deposited collagen and blood vessels, but also for the survival of autologous skin grafts applied on the biomaterial-treated wounds. Only when spacing among granules were ensured, split grafts were capable of surviving and integrating with the surrounding healthy tissue, encouraging both collagen formation and angiogenesis.

The present pre-clinical study clearly shows the tissue regeneration potential of soybean-based biomaterials. The use of these biomaterials present a number of potential advantages including:

1. A degradation time faster than collagen-based biomaterials;[19]
2. An inflammatory response maintaining the neutrophil/macrophage balance in favor of the neutrophils, thus reducing the risks of infections and of a chronic inflammation leading to scarring;[16,19]
3. The stimulation of collagen synthesis and angiogenesis promoting tissue repair in wounds not capable of spontaneous healing.

Together with the relatively simple manufacturing process of these biomaterial and flexible formulations, these properties highlight the clinical potential of these biomaterials that can represent a unique alternative to less performing dressings and dermal substitutes. However, the study also indicates that the clinical outcome of wounds treated by these biomaterials relies on the optimal design of their formulation and correct use by the practitioners.

## New Features on the Journal’s Website

### (1) Advanced online publications (AOP)

Articles accepted will be published online before being included in a printed edition, so it can be available earlier.

### (2) APP for iPad®

A free application to browse and search the journal’s content is available for iPad®. To download the App for iPad®:

- Search “burns trauma” in App Store, or
- Visit the journal’s website, and scan the QR-code in the lower right corner to download the APP.

### (3) News

Breaking news in the field of burns and trauma are reported on the website with short comments occasionally.
